# The BpTRU automatic blood pressure monitor compared to 24 hour ambulatory blood pressure monitoring in the assessment of blood pressure in patients with hypertension

**DOI:** 10.1186/1471-2261-5-18

**Published:** 2005-06-28

**Authors:** Linda Beckett, Marshall Godwin

**Affiliations:** 1 Linda Beckett is currently a 3^rd ^year Family Medicine Resident at the Dept of Family Medicine, Queen's University, Kingston, Ontario, Canada; 2 Marshall Godwin is a Professor, Queen's University and the Director, Centre for Studies in Primary Care, Department of Family Medicine, Queen's University, 220 Bagot Street, Kingston, Ontario, K7L 5E9, Canada

## Abstract

**Background:**

Increasing evidence suggests that ABPM more closely predicts target organ damage than does clinic measurement. Future guidelines may suggest ABPM as routine in the diagnosis and monitoring of hypertension. This would create difficulties as this test is expensive and often difficult to obtain. The purpose of this study is to determine the degree to which the BpTRU automatic blood pressure monitor predicts results on 24 hour ambulatory blood pressure monitoring (ABPM).

**Methods:**

A quantitative analysis comparing blood pressure measured by the BpTRU device with the mean daytime blood pressure on 24 hour ABPM. The study was conducted by the Centre for Studies in Primary Care, Queen's University, Kingston, Ontario, Canada on adult primary care patients who are enrolled in two randomized controlled trials on hypertension. The main outcomes were the mean of the blood pressures measured at the three most recent office visits, the initial measurement on the BpTRU-100, the mean of the five measurements on the BpTRU monitor, and the daytime average on 24 hour ABPM.

**Results:**

The group mean of the three charted clinic measured blood pressures (150.8 (SD10.26) / 82.9 (SD 8.44)) was not statistically different from the group mean of the initial reading on BpTRU (150.0 (SD21.33) / 83.3 (SD12.00)). The group mean of the average of five BpTRU readings (140.0 (SD17.71) / 79.8 (SD 10.46)) was not statistically different from the 24 hour daytime mean on ABPM (141.5 (SD 13.25) / 79.7 (SD 7.79)). Within patients, BpTRU average correlated significantly better with daytime ambulatory pressure than did clinic averages (BpTRU r = 0.571, clinic r = 0.145). Based on assessment of sensitivity and specificity at different cut-points, it is suggested that the initial treatment target using the BpTRU be set at <135/85 mmHG, but achievement of target should be confirmed using 24 hour ABPM.

**Conclusion:**

The BpTRU average better predicts ABPM than does the average of the blood pressures recorded on the patient chart from the three most recent visits. The BpTRU automatic clinic blood pressure monitor should be used as an adjunct to ABPM to effectively diagnose and monitor hypertension.

## Background

Hypertension is a continuous, independent, yet modifiable risk factor for cardiovascular, cerebrovascular and renal disease. It has been estimated that 62% of cerebrovascular disease and 49% of ischemic heart disease can be attributed to suboptimal blood pressure(BP) control [1].

Since 24 hour ambulatory blood pressure monitoring (ABPM) is currently recommended as the measurement of choice in difficult cases (uncertainty of diagnoses, fluctuating office visits, unresponsiveness to treatment, white coat affect(WCE)) [1-5] it is, in effect, the 'final arbiter' or 'gold standard' for the diagnosis of hypertension and the assessment of whether target has been achieved. The generally accepted blood pressure target on ABPM is a daytime average pressure of < 135/85 mmHG [2-4]; the target blood pressure when measured in the doctors office is < 140/90 mmHG.

Increasing evidence suggests that ABPM more closely predicts target organ damage than does clinic measurement. The Ohsama study found that cardiovascular mortality, but not all cause mortality, was more closely predicted by the daytime systolic blood pressure than clinic pressures [6]. ABPM also better predicted cardiovascular events such as MI, CHF, stroke and TIA [4,5] as well as other target organ damage such as ventricular hypertrophy, proteinuria, plasma creatinine, and stroke [5,7-14].

Many patients have clinically significant WCE, making clinic measurements an unreliable indicator of true blood pressure control [11]. Future guidelines may suggest ABPM as routine in the diagnosis and monitoring of hypertension. This would create difficulties as this test is expensive and often difficult to obtain.

Recently, automatic blood pressure measuring devices have been used either at home or in the clinic setting [16]. It has been found that the measurements taken at home, or while not in the presence of clinic staff, have a better correlation with daytime ABPM than do nurse or physician measurements [15-17]. Home or clinic self measurement is also preferred by patients over 24 hour monitoring [18].

The purpose of this study is to determine the possible clinical utility of the BpTRU automated blood pressure monitor in the diagnosis and monitoring of hypertension in the primary care clinic setting. Specifically, we set out i) to determine how BpTRU measurements related to ABPM measurements and ii) to determine the level of BpTRU measurement that best predicted a mean daytime blood pressure of <135 mmHG systolic and < 85 mmHG diastolic. The BpTRU monitor has been developed by VSM MedTech Ltd of Vancouver Canada specifically for the clinician's office.

## Methods

The data used in this study is a subset of the data collected for two ongoing RCTs being conducted at the Centre for Studies in Primary Care at Queen's University, Kingston, Ontario, Canada – the Home Monitoring of Blood Pressure Study (ISRCTN25105161) and the Intensive Scheduled Management of Hypertension Study (ISRCTN05874865) which are funded by the Heart and Stroke Foundation of Ontario. A group of 481 subjects for which all relevant data were available were used for analyses of correlations.

Subjects were recruited from 51 family practices in eastern Ontario. All patients with a diagnosis of hypertension who were being treated with antihypertensive medications were identified in each practice. Each patient's chart was reviewed to abstract the blood pressures recorded on the patient's chart at the last three office visits where blood pressure was measured. Only one recording was used from any single office visit. If there was more than one recording at a given visit, the last measurement recorded for that visit was used. These visits ranged from several weeks to several months apart, depending on the practice of the physician regarding follow-up of hypertensive patients. If the mean of these three readings, taken at different visits, was ≥140/90, the patient was labeled as 'uncontrolled' by office measurement, meaning that the patient had not achieved the treatment target (≥130/80 was used for diabetics). The research nurse then contacted these uncontrolled patients and invited them to participate in the study. Subjects were excluded if they were <18 years of age, pregnant, or had a known secondary cause for their hypertension.

### BpTRU measurements

The BpTRU device uses the oscillometric technique used by most ambulatory and home blood pressure measuring devices [19,20]. It is designed to take an initial reading while the clinician is present, and then with the patient alone in the room, proceeds to take 5 more measurements at intervals of 1–5 minutes and then provides an average of these five readings.

The specific steps we used were: i) the subjects were seated for at least 5 minutes, ii) the BpTRU cuff was applied to the non-dominant arm by the research nurse, iii) the initial BpTRU blood pressure reading was taken and recorded, iv) the staff then left the room while the BpTRU device took a minimum further 5 readings at intervals of either one minute or two minutes, v) these five readings were averaged by the device and this average was recorded. The BpTRU device has passed the standards of the British Hypertension Society and the Association of Advancement of Medical Instrumentation [20,21].

### Ambulatory monitoring

All patients then had ABPM monitoring using oscillometric A&D Model TM2430 equipment (A&D Medical, Milpitas, California, USA). The ABPM equipment was applied at the same visit as the other research measurements. The cuff was fixed to the non-dominant arm and the device was set to obtain automatic readings every 15 minutes during the day (0600–2200) and every 30 minutes at night (2200–0600). This monitoring took place on working days and subjects were instructed to behave and work as usual. The A&D Model TM2430 has been clinically validated according to the British Hypertension Society protocols [22].

## Results

Of the 481 subjects, 210 (43.6 %) were male and 271 (56.3 %) female. The average age was 64.9 (SD 11.59) with a range of 33 to 92 and average BMI was 30.6 (SD 5.22). All but 2 subjects were on antihypertensive medications. In essence, this was a population of treated known hypertensive patients.

### Comparison of group mean blood pressures

Table 1 shows the mean and standard deviations(SD) of systolic and diastolic BP as measured in the family physicians' offices (the average of the blood pressure measurements recorded on the patient chart at the last three visits), the initial BpTRU reading, the average BpTRU reading, and the 24 hour daytime average. There was no statistical difference in group mean between clinic measurement and initial BpTRU readings, and no statistical difference between average BpTRU readings and ABPM daytime average.

**Table 1:** Mean systolic and diastolic BPs

Average of the blood pressures measured at the last three office visits	150.8 (SD10.26) / 82.9 (SD 8.44)
BpTRU initial reading	150.0 (SD21.33) / 83.3 (SD12.00)
BpTRU Average	140.0 (SD17.71) / 79.8 (SD 10.46)
24 hour daytime average	141.5 (SD 13.25) / 79.7 (SD 7.79)

### Comparison of BP target achievement

A large proportion of those subjects who had uncontrolled hypertension according to the mean of the three last clinic BPs had lower blood pressure on either or both of BpTRU or 24 hour monitor. In 393 (81.7%) of the 481 subjects the systolic blood pressure was less with the BpTRU than with the clinic readings and in 312 (64.8%) of the 481 subjects the systolic blood pressure was less with daytime ABPM than with the clinic readings. Diastolic pressure similarly decreased in 332 (69.0%) using the BpTRU average and 292 (60.7%) with daytime ABPM. In all, 250 of the 470 (53.2%) were found by BpTRU monitor to have achieved the accepted clinic measurement target of <140/90 mmHG. ABPM revealed that 162 subjects (33.4%) were actually normotensive with a daytime average of <135/85 mmHG.

### Correlations

Pearson correlations (Table 2) were calculated comparing the mean daytime systolic and diastolic ABPM results with the average of the blood pressure measurements recorded on the patient's office chart, the first BpTRU measurement taken in the presence of the staff, and with the average of five readings on the BpTRU device.

**Table 2:** Pearson Correlation coefficients

	Mean 24 hr daytime systolic	Mean 24 hr daytime diastolic
Mean of the blood pressure measurements at the last three office visits	r = 0.145	r = 0.316
1st BpTRU measurement	r = 0.473	r = 0.554
Average BpTRU(out of 5)	r = 0.571	r = 0.610

### Using BpTRU to predict achievement of targets

ABPM is accepted as the most reliable way to determine if blood pressure targets are achieved in hypertensive patients, but using ABPM after every medication adjustment is impractical and costly. To determine which level of BpTRU blood pressure best predicted achievement of target (i.e. <135/85) on ABPM, various prediction estimates (sensitivity, specificity, and predictive values) were calculated for different levels of BpTRU readings (Tables 3 and 4).

**Table 3:** Two-By-Two Tables And BpTRU Predictive Characteristics For Achievement Of ABPM Systolic Target

		Systolic Target Achieved on ABPM (<135 mmHG mean daytime blood pressure)		
		Yes	NO	

< 140 mmHG on BpTRU	Yes	131	137	Sens = 80%; Spec = 55% PPV = 49%; NPV = 84%
	No	32	170	
< 135 mmHG on BpTRU	Yes	111	101	Sens = 68%; Spec = 67% PPV = 52%; NPV = 80%
	No	52	206	
< 130 mmHG on BpTRU	Yes	86	59	Sens = 53%; Spec = 81% PPV = 59%; NPV = 76%
	No	77	248	
< 125 mmHG on BpTRU	Yes	62	31	Sens = 39%; Spec = 90% PPV = 67%; NPV = 73%
	No	101	276	
< 120 mmHG on BpTRU	Yes	42	12	Sens = 26%; Spec = 96% PPV = 78%; NPV = 71%
	No	121	295	

**Table 4:** Two-by-Two Tables and BpTRU predictive characteristics for achievement of ABPM diastolic target

		Diastolic Target Achieved on ABPM (<85 mmHG mean daytime blood pressure)		
		Yes	NO	

< 90 mmHG on BpTRU	Yes	341	58	Sens = 92%; Spec = 43% PPV = 86%; NPV = 61%
	No	28	43	
< 85 mmHG on BpTRU	Yes	299	36	Sens = 81%; Spec = 64% PPV = 89%; NPV = 48%
	No	70	65	
< 80 mmHG on BpTRU	Yes	238	13	Sens = 65%; Spec = 87% PPV = 95%; NPV = 40%
	No	131	88	
< 75 mmHG on BpTRU	Yes	161	5	Sens = 44%; Spec = 95% PPV = 97%; NPV = 32%
	No	208	96	
< 70 mmHG on BpTRU	Yes	82	1	Sens = 22%; Spec = 99% PPV = 99%; NPV = 26%
	No	287	100	

## Discussion

The measurements of blood pressure done with a provider present, that is the mean of the physician office measurements, and the observed first BpTRU measurements were not significantly different from each other and probably are both affected by the white coat phenomenon. This also suggests that the mean of the last three office blood pressure measurements is a good proxy for the patient's current blood pressure, taken with a health care professional present (an observed blood pressure measurement). Both of these measures, that is, the mean of the last three office blood pressures and the first, observed reading from the BpTRU, were significantly higher than multiple measurements taken when a provider is not present, that is the mean of the five BpTRU measurements and the daytime mean on ABPM. The mean of the BpTRU and the daytime mean on ABPM were not significantly different.

### Recommended BpTRU Thresholds

Our data suggest that the degree of agreement between the BpTRU and 24 hour ABPM is not sufficient for clinicians to use BpTRU alone to determine if BP targets have been achieved. However, since the agreement between the BpTRU and ABPM is so much better than the usual sphygmomanometer-based, observed, clinic measures, it seems reasonable to use the BpTRU to make treatment adjustment decisions to a predetermined BpTRU level and then confirm it with a 24 hour ABPM. If one considers Table 3, it would seem that a systolic BP level of <135 mmHG provides the best overall agreement with the 24 hour ABPM. Similarly, for the diastolic two-by-two tables in Table 4, one would likely choose <85 mmHG diastolic as the most appropriate BpTRU level for diastolic pressure.

Clinicians should consider treating patients to a level of <135/85 mmHG using the BpTRU or similar automated multiple reading device, and at that point conduct a 24 hour ABPM to confirm that a daytime mean pressure of <135/85 mmHG has been achieved. If it has not been achieved, the clinician should further treat to a BpTRU level of <130/80 mmHG or <125/75 mmHG before re-assessing with 24 hour ABPM.

### Limitations

Our data are the best currently available to provide some sense of what a BP target level should be when using the BpTRU in office practice. However there are some limitations. The BpTRU and ABPM were carried out in a research setting not a true clinical setting. All the patients had uncontrolled blood pressure according to office BP measurements, so it does not represent a full spectrum; it does not include normotensive patients or patients where the chart records suggest target has been achieved. The data was collected in the course of another study. There is a need for a study that sets out, a priori, to compared BpTRU with ABPM in the full spectrum of patients, and which follows those patients for a longer term to assess clinical outcomes.

## Conclusion

The control of hypertension is vital to decrease cardiovascular morbidity and mortality, however the current recommended BP targets are often not being met. There is also growing evidence that office measured sphygmomanometer-based blood pressures are unreliable and do not predict outcomes as well as ABPM, whereas there is increasing evidence from prospective trials that 24 hour monitoring has prognostic significance. We have shown that the BpTRU has potential to be used in the clinic setting to help overcome the difficulties caused by the WCE without the cost of having to conduct frequent 24 hour ABPMs.

Although this study demonstrates that the BpTRU has a sensitivity and specificity that are not ideal when compared to the ABPM device we used, it is superior to usual office measurement and can be used by clinicians as part of their strategy for determining whether BP target has been achieved. We suggest that the BpTRU be used to adjust treatment until patient's BpTRU pressure is below 135/85 mmHg and then a 24 hour ABPM be conducted to confirm. ABPM measurement is not practical as a means of monitoring targets after each medication adjustment but in conjunction with the BpTRU can form the basis for clinical decisions that will promote more effective control of hypertension.

## Competing interests

The author(s) declare that they have no competing interests.

## Authors' contributions

LB abstracted the data from the larger baseline dataset from the randomized controlled trials. She analysed the data, did the initial interpretation and wrote the initial draft of the article.

MG designed and is conducting the randomized controlled trials from which the data for this study was drawn. He worked with LB on the analysis and the data interpretation and helped with the substantial re-writing of the original draft of the manuscript.

## Pre-publication history

The pre-publication history for this paper can be accessed here:


